# Urban and Wild Kelp Gulls: Tracking Seasonal Shifts in Habitat Use and Movement Patterns

**DOI:** 10.1002/ece3.72974

**Published:** 2026-01-20

**Authors:** Miriam Lerma, Mylene Seguel, Claudia E. Fernández, Stefan Garthe, Guillermo Luna‐Jorquera

**Affiliations:** ^1^ Research and Technology Centre (FTZ) University of Kiel Büsum Germany; ^2^ Departamento de Biología Marina, Facultad de Ciencias del Mar Universidad Católica del Norte Coquimbo Chile; ^3^ Laboratorio de Investigación de Ciencias Veterinarias, Facultad de Ciencias Agropecuarias Universidad del Alba La Serena Chile; ^4^ Escuela de Ciencias Biológicas Universidad Nacional Heredia Costa Rica; ^5^ Centre of Ecology and Sustainable Management of Oceanic Islands (ESMOI), Facultad de Ciencias del Mar Universidad Católica del Norte Coquimbo Chile

**Keywords:** anthropogenic, gull, seabird, synanthropic, tracking, urban

## Abstract

Humans impact the environment through a myriad of activities, including urbanisation. While some wildlife species struggle to cope with urban development, others might benefit from it. Kelp gulls ( *Larus dominicanus* ) population growth has been linked to the availability of anthropogenic resources; however, there is limited knowledge about their habitat use and movement patterns year‐round. Here, we tracked Kelp gulls (*n* = 8) in Northern Chile from December 2022 to December 2023 using GPS‐GSM devices. Our goal was to gain understanding of their habitat use and movement patterns throughout the year, and to test for differences between an urban colony at Coquimbo and a wild colony in Damas Island. We found that both wild and urban gulls used natural habitats such as the open sea and wetlands, as well as anthropogenic habitats, such as landfills and fish markets. Wild gulls undertook longer trips and travelled greater distances than urban gulls, and this was attributed to wild gulls' colony being farther from predictable food resources. In addition, there were differences in habitat use and movement patterns across the seasons. During the breeding period, wild gulls use anthropogenic habitats to a lesser extent than urban gulls, but during the post‐breeding period, wild gulls ventured farther from their colonies to more anthropogenic habitats. Although based on a limited sample size, we found that urban gulls behave as synanthropic animals, showing reduced mobility year‐round, whereas wild gulls' movements suggest that they are adapting to shifts in their breeding responsibilities and to variations in food availability. Beyond contributing to gull ecology, these findings provide valuable information for conservation and management planning.

## Graphical abstract

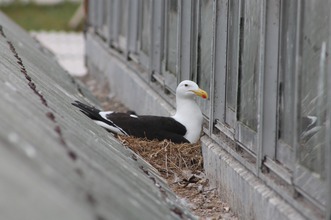

## 1 Introduction

Humans are causing profound impacts on the environment through a myriad of activities including urbanisation. For wildlife, some species show greater tolerance towards anthropogenic changes than others (McKinney and Lockwood 1999; Ouled‐Cheikh et al. 2021). For many seabirds, global changes have caused adverse effects on their populations leading to many species being classified as critically endangered, endangered or vulnerable (Croxall et al. 2012). The decline in population is particularly intense for specialist species, as they are often not flexible enough to adapt to the fast ecological changes in their habitats. In contrast, opportunistic and scavenging species populations are growing in several parts of the world, as anthropogenic ecological changes offer them advantages (Lisnizer et al. 2011; Navarro et al. 2017; Ouled‐Cheikh et al. 2021). This is the case for many species of gulls, which are opportunistic feeders, breeders, or both (Oro et al. 2013; Yoda et al. 2012). However, the increasing numbers of large gulls lead to conflicts, such as the transportation of pathogens or pollution (Fernández et al. 2024; Ghersi et al. 2009; Pereda et al. 2008; Rodríguez et al. 2012; Yorio et al. 2024), as well as an increased pressure on threatened species by predation (Ludynia et al. 2005; Pastén‐Araya et al. 2021; Simeone and Luna‐Jorquera 2012). Moreover, although the food resources of human origin are often assumed to improve breeding and promote population growth (Oro et al. 2013; Serré et al. 2022; Svagelj et al. 2015; Washburn et al. 2016), human waste can also have negative impacts on gulls' survival, health, and breeding success (Adami et al. 2024; Canti et al. 2023; Pierotti and Annett 2001; Yorio et al. 2014).

**FIGURE 1:**
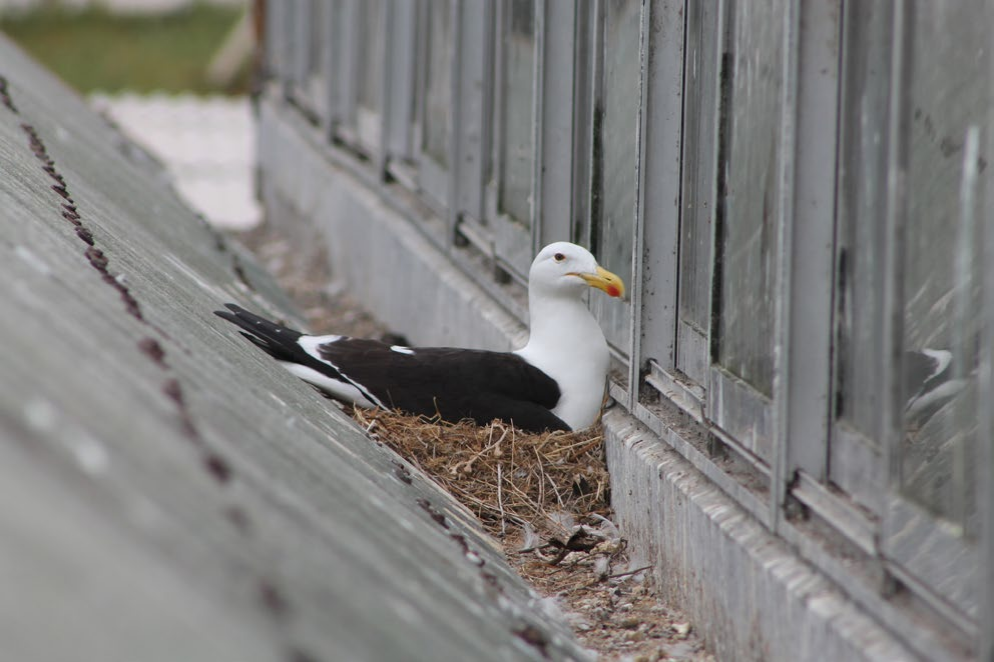
Kelp gull ( *Larus dominicanus* ) nesting on a rooftop in Coquimbo. Photo by C. E. Fernández.

Kelp gulls ( *Larus dominicanus* ) (Figure 1) are widely distributed throughout much of the Southern Hemisphere (Yorio et al. 2016). This gull species is opportunistic and omnivorous, capable of scavenging as well as actively seeking prey in a wide variety of habitats (Bertellotti and Yorio 1999; Kasinsky et al. 2018; Reusch et al. 2020; Silva‐Costa and Bugoni 2013; Steele 1992; Yorio et al. 2024). In Chile, some Kelp gull colonies are formed on rooftops in highly urbanised cities (Chávez‐Villavicencio 2014), whereas other colonies continue to breed in remote wild areas, such as protected islands with no human presence (Yorio et al. 2016). In other gull species inhabiting urban areas, it has been found that they rely to a greater extent on resources within the cities, whereas in wild areas, they rely to a greater extent on marine resources found in coastal areas or the open sea (Langley et al. 2022; Pierotti and Annett 2001; Reusch et al. 2020). Moreover, it has been found that due to the differences in the predictability and abundance of the food resources, gulls in urban areas travel directly to areas that have a more constant food access (Plaza and Lambertucci 2017; Yorio et al. 2024), whereas gulls in wild areas tend to exhibit more dispersed movement patterns (Yoda et al. 2012). However, habitat use and movement patterns are often studied only during the breeding period, while much less is known about the post‐breeding period. Yet, the predictability and abundance of food resources vary throughout the course of the annual cycle, as do the birds' energy requirements, which are typically higher during the breeding period (Bertellotti and Yorio 1999; Hamer et al. 2002; Whittington et al. 2009). As a result of this resource fluctuation and shifting energy requirements, variation in habitat use and movement patterns over their annual cycle is commonly observed in other species of gulls (Frixione et al. 2023; O'Hanlon et al. 2022; Ramírez et al. 2020).

Here, we present the first study of Kelp gulls using solar‐powered GPS–GSM technology. Such long‐term deployment provides one of the first year‐round datasets on habitat use and movement patterns of Chilean Kelp gulls, offering new insights into their ecology beyond the breeding period. In this study, we define *urban gulls* as those nesting in human‐influenced areas, where anthropogenic resources are abundant and located near their colonies, and *wild gulls* as those nesting in natural areas with minimal or no interaction with human‐derived resources. Specifically, urban gulls at Coquimbo nest close to large fishing ports and major municipal landfills, which might offer constant and predictable resources. In contrast, wild gulls from Damas nest within the protected Punta de Choros Marine Reserve, close to highly productive but seasonally variable upwelling systems and to artisanal fishery discards, making the resource dichotomy particularly relevant in this region. We expect that (1) urban Kelp gulls in Chile will visit anthropogenic habitats in greater proportion than wild gulls, whereas wild gulls will visit a greater proportion of natural habitats, (2) urban gulls will remain closer to their colonies and travel shorter distances compared to wild gulls due to the proximity to more abundant and predictable resources, and (3) urban and wild gulls will adjust their habitat use and movement patterns throughout the year in response to environmental changes and shifting energy budgets between the breeding and the post‐breeding periods.

## 2 Methods

### 2.1 Study Site

**FIGURE 2:**
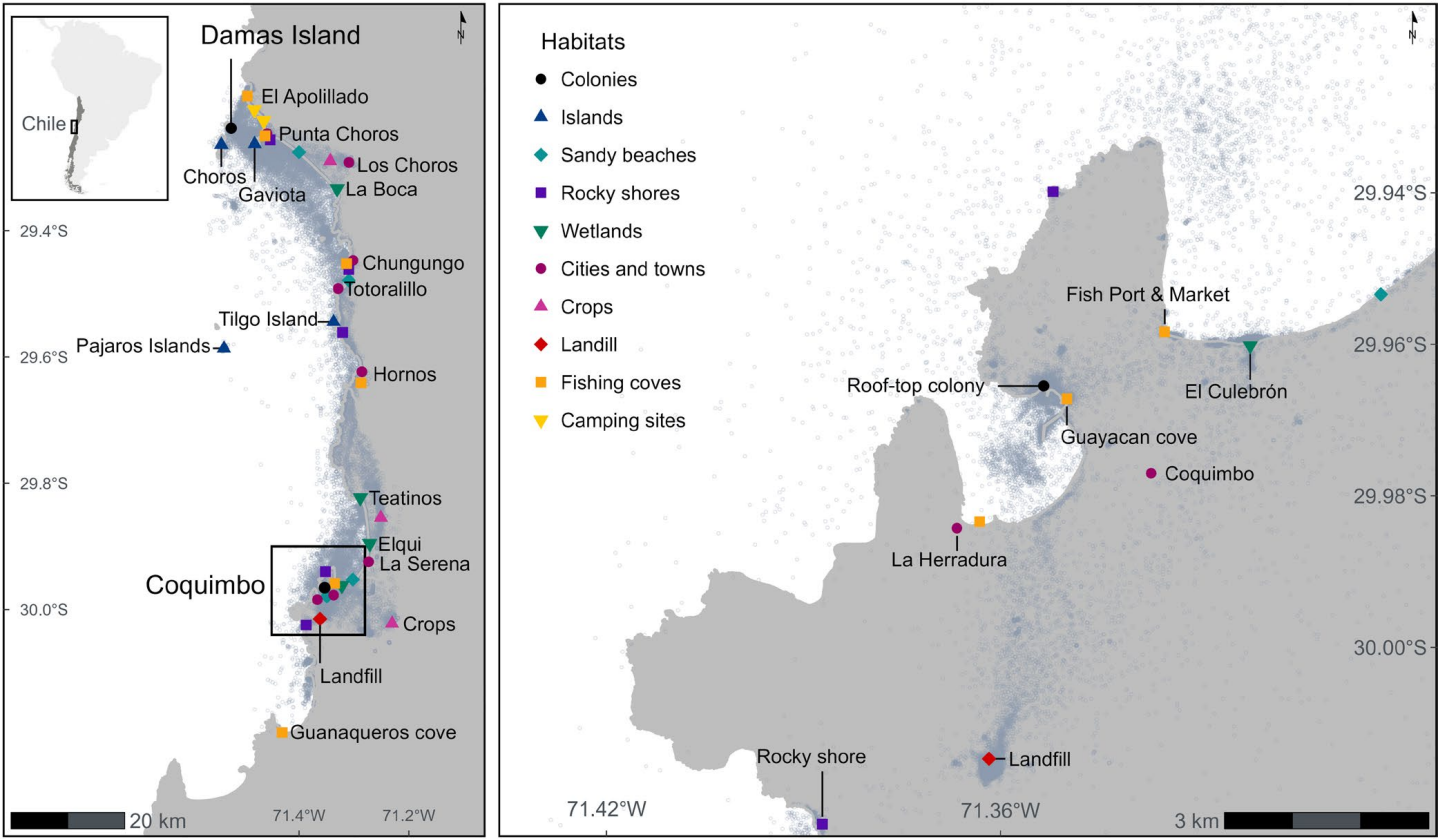
Kelp gulls from two colonies were studied: gulls nesting on Damas Island were classified as *wild gulls* and gulls nesting in Coquimbo were classified as *urban gulls*. Grey dots represent tracking locations recorded during the study period (December 2022–December 2023). Main habitat types are represented by different shapes and colours, with the important sites names labelled.

Fieldwork was conducted in northern Chile, where two colonies were studied. The first colony is located on the rooftop of a university building in Coquimbo City (29° 57′ S, 71° 20′ W), whereas the second colony is situated on the uninhabited Damas Island (29° 13′ S, 71° 31′ W) (Figure 2). In the rooftop colony, a total of 35 breeding pairs were estimated between November 2021 and March 2022 (Fernández *pers. comm*.). However, additional roofs are occupied by gulls, and estimating the total number of pairs across buildings is difficult due to their inaccessibility. Damas Island colony is estimated at approximately 100 pairs (Simeone et al. 2003). However, new censuses on Damas Island are needed for updated estimates.

Damas Island is part of a marine protected area, has an area of 56 ha, and is located 5.2 km away from the mainland. Urban gulls breeding in the rooftops of Coquimbo nest approximately 80 km away from the wild gulls breeding on Damas Island. In the Coquimbo region, which includes Coquimbo, La Serena and Damas, upwelling events are concentrated during the austral summer and spring (Thiel et al. 2007). These upwelling events support zooplankton and fish production over extensive areas, which in turn sustain large populations of seabirds and marine mammals (Thiel et al. 2007). Fishing coves and markets are spread along the coastline and represent one of the region's main economic activities. Higher landings and by‐catch coincide with the period of upwelling (Hernández et al. 2010). Besides fishing, tourism is another major economic activity in the region. Coquimbo, La Serena and the area of Damas are well‐known tourist destinations in Chile (Balaguera‐Quintero et al. 2022), attracting a high number of visitors during summer (Simeone et al. 2003). Agriculture is also an important economic activity in the region, and olive cultivation, for example, is widespread. In terms of urbanisation, La Serena‐Coquimbo is one of the fastest‐growing urban areas in Chile (Orellana McBride 2020). The region relies on a single open landfill, which receives hundreds of tons of waste generated daily (Balaguera‐Quintero et al. 2022).

### 2.2 Data Collection

A total of nine Kelp gulls of unknown sex were captured between October and December 2022. Fieldwork was performed following ethical international standards for the care and use of wild animals from Ministerio de Agricultura (SAG), resolución exenta No. 28/2022, and approved by the Universidad Católica del Norte's Scientific Ethics Committee by resolution CEC UCN No. 46.

Gulls were captured using walk‐in wire mesh traps (50 × 50 × 75 cm) with a string trigger set on top of nests containing eggs. Once captured, each gull was marked with a colour plastic ring, weighed, and measured for wing length (398–430 mm) and tarsus (77–81 mm). Tracking devices were deployed using a body harness backpack made of two Teflon strings, secured with aluminium crimps, knots, and glue (Thaxter et al. 2014). The devices deployed were Global Positioning System‐Global System for Mobile communication (GPS‐GSM) (OrniTrack‐30, Ornitela, Lithuania). The device weight, including the harness, was approximately 22 g, well below the 3% weight threshold (Kelp gulls' weight: 800–1000 g).

Research on tracking device effects on gulls has yielded conflicting results: some studies suggest negative effects on breeding success, while others find no indication of adverse effects (Souc et al. 2024). For the period of the study, only one gull was lost. The lost gull carried the device only for 1 month until 25 December 2022. This gull was found dead near the fish market with a fishing hook piercing its bill. The cause of death could not be linked to the effect of the tracking device.

As of November 2025, five of the nine gulls are still sending data. After the period of this study (December 2023), two devices were lost. Based on the accelerometer data, the harnesses loosened and fell off while the gulls were roosting, one on 3 March 2024 on a Coquimbo rooftop, and another one on 10 March 2025 in Totoralillo. An additional gull was lost on 1 February 2024, as it was euthanized by the park authorities in Damas Island to prevent the spread of avian flu, as it was suspected to be infected. Due to the restricted access to the colonies caused by the avian flu outbreak, we were unable to evaluate the potential adverse effects of the devices on the tracked birds. Nevertheless, the observation that the tracked gulls returned to their colonies to breed in subsequent seasons suggests no clear negative effects of the devices. Moreover, similar devices have been previously used on this gull species without raising concerns about behavioural impacts (Reusch et al. 2020).

### 2.3 Data Manipulation

Tracking data was restricted to the period between the 3 of December 2022 to 3 of December 2023. This period represents exactly 1 year following the last deployment. Austral summer comprises December, January, and February, coinciding with the breeding season of Kelp gulls. This includes incubation, which lasts about 27 days (Prellvitz et al. 2009; Yorio and García Borboroglu 2002), and the chick rearing period, during which chicks become fledglings at approximately 7 weeks old (Prellvitz et al. 2009; Yorio and García Borboroglu 2002). Autumn included the months of March, April, and May, and encompasses the post‐fledgling period, when adults are followed by juveniles, until the latter reach around 14 weeks old (Prellvitz et al. 2009). Winter comprises June, July, and August, and represents the post‐breeding period, when birds are free from breeding responsibilities. Spring comprises September, October, and November, and includes the transition between post‐breeding period and the pre‐breeding period.

The GPS‐GSM devices were powered by solar panels, allowing continuous operation without the need for battery replacement or retrieval. To ensure long‐term functionality, the devices were programmed to adjust their recording intervals based on battery charge. Of all GPS fixes collected during the study period: 38% were recorded every 5 min (full battery charge); 41% every 15 min (charge > 75%); 12% every 30 min (charge < 75%); 5% approximately every hour (charge < 50%); and less than 2% were recorded less than once per hour (charge < 25%). Seasonal changes influenced the recording frequency: favourable sunlight conditions allowed for optimal battery charge and more frequent GPS fixes during austral summer (December–February). In contrast, reduced sunlight in winter (June–August) resulted in lower battery charge levels and less frequent recording intervals.

Trips were identified using the R package ‘larus’ (Lerma 2025). A trip was defined as an individual's movement at least 300 m away from it's central location, and remaining away for more than 30 min. The precision of OrniTrack devices has been estimated around 20 m (Clements et al. 2021). During the breeding period, the central location was the colony. During the non‐breeding period, the central location was the colony or a resting location. Resting locations were identified as clusters of locations where the gulls overnighted or stayed > 24 h. Trips were visually inspected, and trips with unlikely locations and those with speeds exceeding 70 km/h or outliers were removed. Due to differences in sunlight conditions and battery charge, more trips were identified in summer (*n* = 1281) and autumn (*n* = 847) than in winter (*n* = 292) and spring (*n* = 519).

### 2.4 Habitat Use

Using the locations of the tracked individuals, different habitat types were identified, and spatial polygons were manually created in QGIS 3.28. The manual delineation of the habitats was guided by the habitat classification provided by the Google Maps Street View layer, which identifies features such as wetlands, sandy beaches, fishing ports, landfills, and agricultural fields. Polygons were digitised in QGIS using ‘Add Vector Layer’, following boundaries observed in satellite imagery. For fishing markets, coves and ports, an area of influence for around 1 km offshore from the dockside or coastline was included to account for fish remains thrown back at sea, either from the gutting process at port (Banegas Vizuete et al. 2018), or directly from fishing vessels (Villablanca et al. 2007). Additionally, the coastal influence area for these ports extended 200 m inland to include immediate processing facilities and waste disposal points. Agricultural fields were delineated by tracing the boundary of actively cultivated parcels visible in the satellite imagery, ensuring that only clear farming areas were included. Urban and suburban areas were defined as all built‐up environments and associated infrastructure (e.g., roads, buildings), using the boundaries of contiguous high‐density development. To confirm a correct habitat classification, sites that were accessible were directly visited.

Habitats were classified as natural habitats (coastal areas, the open sea, wetlands, rocky shores, sandy beaches and uninhabited islands) or anthropogenic habitats (areas with human presence, including coastal transformed areas such as urban and suburban areas, landfills, agricultural fields, recreational beaches or camping areas frequented by tourists and locals, and fish markets, coves or ports). To account for differences in temporal resolution, prior to the calculation, the locations considered trips were linearly interpolated to an interval of 15 min using the R package ‘larus’ (Lerma 2025). Locations were interpolated for trips lasting less than 24 h and without gaps exceeding 60 min, except for urban gulls during winter. For urban gulls during winter, a threshold of < 90 min gap was allowed, due to reduced light exposure and lower battery levels during this period. From a total of 89,244 original trip locations, the interpolation returned 53,876. The locations were overlaid on the polygons of habitat type, and each location was assigned a habitat type. To quantify the habitat use per bird, as with other gull species (Navarro et al. 2017), the number of habitat‐type positions was divided by the particular period. We acknowledge that reducing fixes through linear interpolation biases habitat use estimates by smoothing short trips and underrepresenting brief or fine‐scale movements, leading to underestimation of localised habitat use and overemphasis of broader‐scale patterns.

### 2.5 Movement Patterns

For each trip, trip duration (h), maximum distance from the colony (km), and the path length (km) were calculated using the package ‘larus’ (Lerma 2025). Trip duration (h) was defined as the total time between departure and return to the central location. Maximum distance from the colony was measured as the most distant point in a straight line from the colony during a trip, regardless of whether the gulls changed central location. Path length was the sum of distances between consecutive fixes within a trip.

### 2.6 Statistical Analyses

To assess differences in habitat use and movement parameters between urban and wild gulls, we used generalised linear mixed models (GLMMs). For habitat use, we fitted models using the proportion of anthropogenic habitat use as a response variable with the package *glmmTMB* (McGillycuddy et al. 2025), specifying a beta distribution with a logit link. For movement parameters (trip duration, maximum distance, and path length), we fitted the models using the package *lme4* (Bates et al. 2015), specifying a gamma distribution. Models included bird status (wild and urban) and season (spring, summer, autumn, and winter) as fixed factors, as well as the two‐way interaction (status × season), with individual identity as a random factor to account for repeated measurements and avoid pseudo‐replication. Model assumptions were examined using the R package *DHARMa* (Harting 2024) for habitat use, and *performance* (Lüdecke 2024) for movement parameters. Models were compared to null models to evaluate significance using type‐III analyses of variance using the package *car* (Fox and Weisenberg 2024). Differences in movement parameters were further compared using Tukey's test with the package *multcomp* (Hothorn et al. 2023). The significance of model parameters was assessed using an *α* value of 0.05. We acknowledge that including multiple fixed effects with only nine individuals increases the risk of overfitting. While GLMMs allow accounting for individual variation, the small sample size may limit the generalizability of the results and inflate the uncertainty of effect estimates. Consequently, effect sizes should be interpreted cautiously, and the model primarily highlights general patterns rather than definitive relationships.

## 3 Results

### 3.1 Habitat Use

**FIGURE 3:**
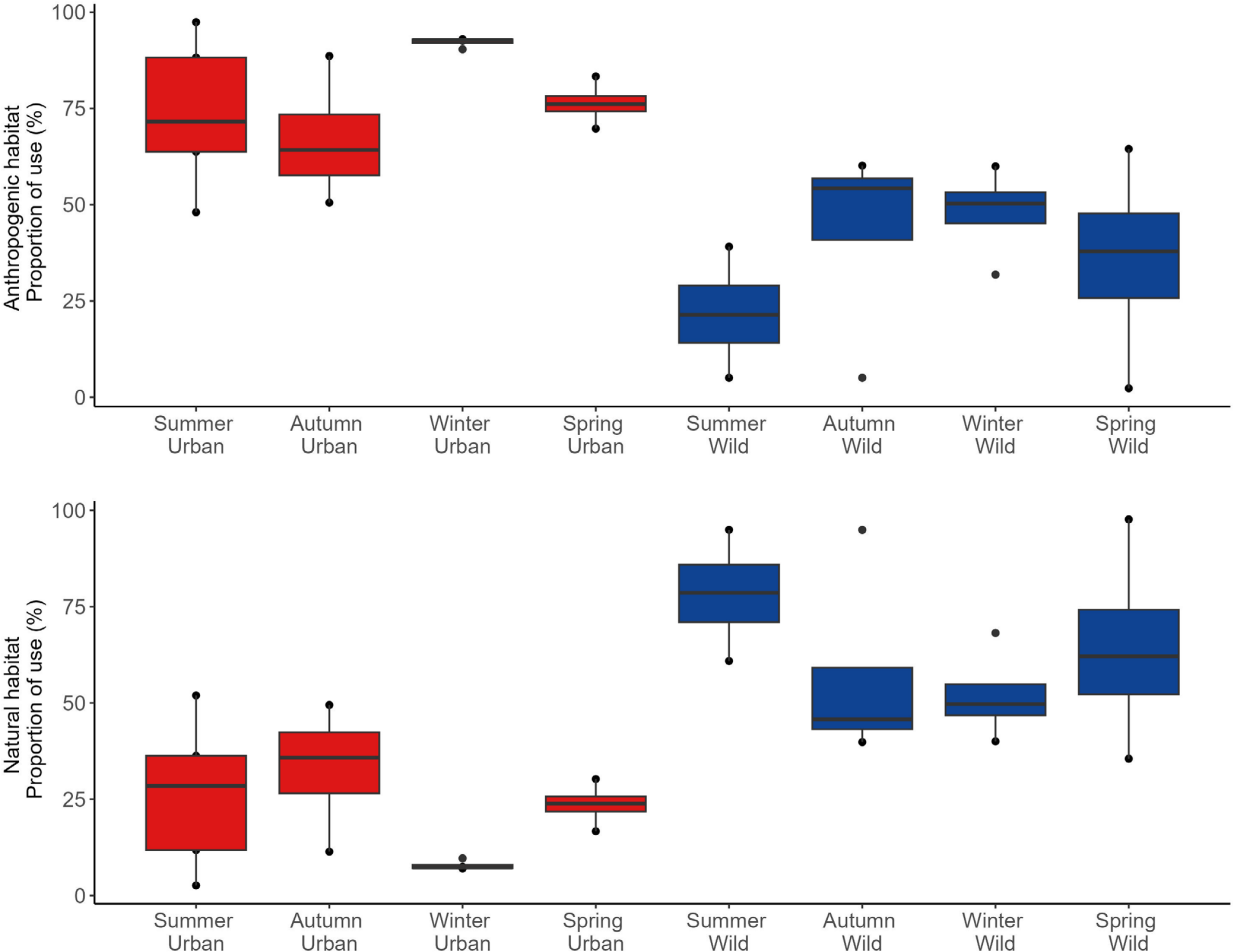
Proportion of anthropogenic (upper panel) and natural (lower panel) habitat use by urban (in red) and wild (in blue) Kelp gulls separated by Austral seasons: Summer (December, January, February), autumn (March, April, May), winter (June, July, August) and spring (September, October, November). Habitat use was calculated as the proportion of locations in each habitat type grouped by season. Box plots show the interquartile range (IQR) across individuals; the horizontal line indicating the median; whiskers extend to 1.5 times the IQR; points represent outliers.

Kelp gulls visited natural and anthropogenic habitats (Figure 2). The interaction between gull type (urban vs. wild) and season was significant in explaining the variation in anthropogenic habitat use (*χ* ^2^ (_1__,__3_) = 12.45, *p* < 0.01) (Figure 3). Urban gulls spent a significantly greater proportion of their time in anthropogenic habitats compared to wild gulls (*χ* ^2^ (_1__,__3_) = 4.13, *p* = 0.04), and the proportion of anthropogenic habitat used varied significantly between the seasons (*χ* ^2^ (_1__,__3_) = 67.83, *p* < 0.01). Overall, urban gulls consistently visited a higher proportion of anthropogenic habitats across all seasons (estimated marginal means 0.66–0.92, 95% CIs above or near 0.50), while wild gulls visited a lower proportion of anthropogenic habitats (0.24–0.48), with summer and spring significantly below 0.50. These results are supported by the observed proportion of sites used. Although urban gulls primarily used anthropogenic habitats year‐round, gulls increased their use of natural habitats, such as the open sea and wetlands, during the breeding period (Figure S1). In contrast, during the post‐breeding period, urban gulls relied almost exclusively on anthropogenic habitats such as city streets, fishing coves and markets, and the landfill (Figure S1). Similarly, wild gulls were primarily using natural habitats, such as the open sea, during the breeding period (Figure S1). However, during the post‐breeding period, wild gulls showed a more pronounced shift than urban gulls by increasing their use of anthropogenic habitats, such as the landfill and fishing coves (Figure S1).

### 3.2 Movement Patterns

**FIGURE 4:**
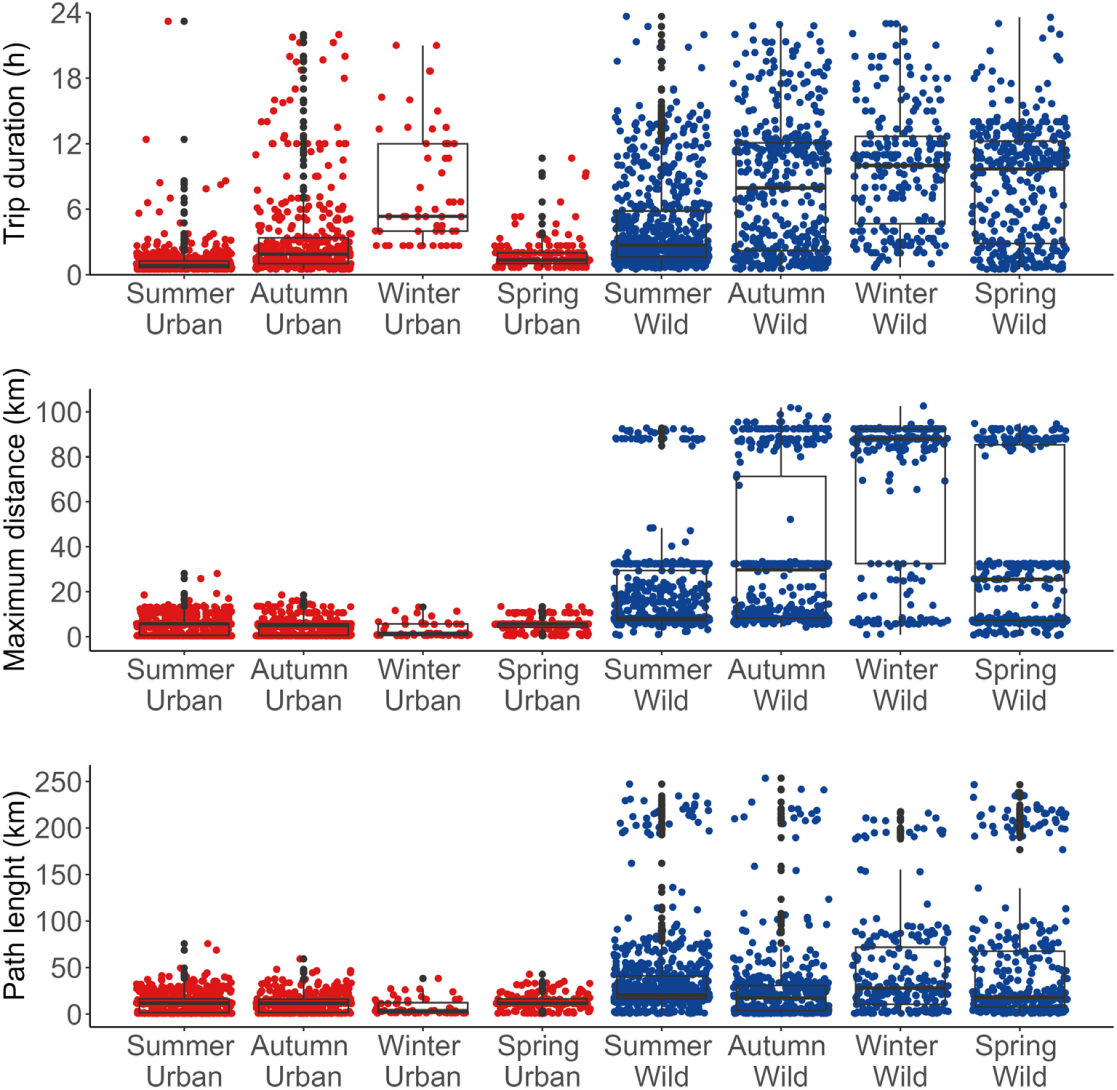
Trip duration (h), maximum distance from the colony (km), and path length (km) of urban (in red) and wild (in blue) Kelp gulls separated by austral seasons. Trip duration is the time between departure and arrival to the colony or resting location. Maximum distance is the farthest point from the colony per trip. Path length is the total distance travelled. Points represent individual trips. Box plots show the interquartile range (IQR) across trips; the horizontal line indicating the median; whiskers extend to 1.5 times the IQR.

The interaction between gull type (urban vs. wild) and season was significant for trip duration (GLMM *χ* ^2^ (_1_,_3_) = 776.0, *p* < 0.01), maximum distance from the colony (GLMM *χ* ^2^ (_1_,_3_) = 44.5, *p* < 0.01), and path length (GLMM *χ* ^2^ (_1_,_3_) = 44.4, *p* < 0.01). Urban gulls consistently made shorter trips than wild gulls across seasons, particularly during summer (Figure 4). Summer trip durations were 0.8 h (95% CI: 0.6–1.3) for urban gulls and 2.7 h (95% CI: 1.6–5.8) for wild gulls (Table S1). Urban gulls also consistently stayed closer to their colonies than wild gulls (Figure 4), but maximum distance from the colony varied seasonally (Table S1), with urban gulls staying closer to their colonies during winter (4 km, 95% CI: 0.6–5.6) and wild gulls during summer (8.1 km, 95% CI: 7.3–29.4). Path length followed a different pattern; urban gulls covered shorter path lengths than wild gulls (Figure 4), with differences among seasons (Table S1). Urban gulls covered shorter path lengths in winter (3.6 km, 95% CI: 1.4–12.3) and wild gulls in autumn (17.4 km, 95% CI: 3.7–30.6).

## 4 Discussion

### 4.1 Habitat Use

Although based on a limited sample size, our findings support the opportunistic nature and flexible behaviour of the Kelp gull, as they visited a wide range of habitats, including both natural and anthropogenic habitats. This result is consistent with previous research on the species (Bertellotti and Yorio 1999; Kasinsky et al. 2018; Reusch et al. 2020; Silva‐Costa and Bugoni 2013; Steele 1992; Yorio et al. 2024). Also, in line with our expectations, urban gulls visited anthropogenic habitats more frequently than wild gulls. This result confirms that Kelp gulls in urban areas rely more heavily on anthropogenic resources, whereas gulls in wild areas rely more heavily on natural resources, as found in South Africa and Argentina (Bertellotti and Yorio 1999; Reusch et al. 2020).

Notably, the observation that wild gulls regularly visited anthropogenic habitats contrasts with findings from two decades ago, when Kelp gulls from Damas Island did not feed on waste that could be associated with the landfill (Ludynia et al. 2005). On one hand, this shift in habitat use by wild gulls might signal degradation of natural habitats. In South Africa, greater use of the open sea by Kelp gulls was attributed to seemingly profitable natural habitats (Reusch et al. 2020). However, the waters around Damas Island are now considered overexploited (Hernández et al. 2010; Thiel et al. 2007). As a result, Kelp gulls might be supplementing their diet with human refuse as an adaptation to less natural prey being available, as occurs in other gull species (Galbraith et al. 2025; Pierotti and Annett 2001; Serré et al. 2022; Whittington et al. 2016). On the other hand, a greater reliance on landfill waste by wild gulls now than before might reflect the growing volume of refuse in the region. La Serena‐Coquimbo is among the fastest‐growing urban areas in Chile (Orellana McBride 2020), and increasing amounts of waste are deposited in the local landfill (Balaguera‐Quintero et al. 2022). To determine whether the observed effect is attributable to reduced prey availability, increase in alternative resources, or both, follow‐up studies on diet and/or stable isotopes are necessary for examining temporal changes in prey abundance and shifts in their incorporation into the gulls' diet.

### 4.2 Movement Patterns

As expected, urban gulls made shorter trips, stayed nearer to their colonies, and covered shorter path lengths than wild gulls. The shorter distances are likely due to urban gulls learning to travel directly to the anthropogenic places near their colonies, where food is predictable and consistently available (Yoda et al. 2012; Yorio et al. 2024). In contrast, wild gulls travelled farther from their colonies and covered longer paths, suggesting a reliance on more variable and less predictable resources. These findings align with studies from South Africa and Argentina, where Kelp gulls nesting closer to anthropogenic resources travelled less far from their colonies (Kasinsky et al. 2018; Reusch et al. 2020) and with synanthropic constraints in urban wildlife (Rodewald and Shustack 2008).

By travelling shorter distances to predictable food resources, urban gulls might enhance efficiency by minimising energetic expenditure, thereby promoting increased survival and reproductive success (Foley et al. 2025; Soriano‐Redondo et al. 2021). Given this advantage of nesting in urban areas, it is unclear why gulls from Damas Island did not intend to nest in urban environments. One possible explanation is that urban gulls maintain year‐round presence on the rooftop colony to defend and preserve existing territories (Quintana and Yorio 1998), potentially preventing new individuals from settling. The Coquimbo rooftop is small (0.01 km^2^) and may have reached their maximum density. Alternatively, urban conditions are not inherently beneficial for all gulls. Wild gulls might prefer nesting closer to their natural habitats to avoid relying on potentially lower‐quality anthropogenic food resources, which could negatively affect their health and reproductive success (Adami et al. 2024; Pierotti and Annett 2001). Additionally, areas far from urban environments might reduce their exposure to anthropogenic risk, including injury or death (Canti et al. 2023; Yorio et al. 2014). In support of this, we report that one of our urban gulls was found with a fishing hook pierced through its bill, exemplifying the exposure to anthropogenic risks while visiting anthropogenic habitats.

### 4.3 Seasonal Differences

As predicted, urban and wild gulls show contrasting habitat use and movement patterns between the seasons. Variation in habitat use and movement patterns throughout the annual cycle is common in gulls (Frixione et al. 2023; O'Hanlon et al. 2022; Ramírez et al. 2020), and it is often attributed to shifting energy requirements and variations in resource availability.

During the breeding period, Kelp gulls remained closer to their colonies as expected for a central place forager. By remaining close to the colony, Kelp gulls likely maximise their time spent incubating, defending and provisioning their chicks (Marinao et al. 2018). Moreover, both urban and wild gulls increased their proportional natural habitats, such as the open sea, during this period, suggesting a preference for marine resources. Many large gulls species shift to a fish‐based diet during breeding, as fish provides higher‐quality nutrients that are essential for egg production (Svagelj et al. 2015) and for chick provisioning (Bertellotti and Yorio 1999). The use of marine resources during breeding also aligns with upwelling events, which are known to increase prey availability (Thiel et al. 2007), and with periods of higher landings and by‐catch reported by fishermen (Hernández et al. 2010). Kelp gulls may take advantage of marine resources either by directly foraging at the open sea, or by scavenging on fishing discards (Giaccardi and Yorio 2004; Kasinsky et al. 2018; Villablanca et al. 2007). Although direct observations would enhance our understanding of the factors driving the gulls to these areas, the use of the marine resources and fishing discards by breeding Kelp gulls is consistent with studies in Argentina (Frixione et al. 2023; Marinao et al. 2018), South Africa (Steele 1992), and Uruguay (Lenzi et al. 2019).

During the post‐breeding period, there were contrasting patterns between urban and wild gulls. Urban gulls remained close to their colonies, aligning with what is commonly observed in animals living in urban areas. Synanthropic animals tend to have smaller territories (Rodewald and Shustack 2008), reduced mobility, and in some cases, a suppressed migration behaviour (Flack et al. 2016). In contrast, wild gulls moved away from their colonies and roosted close to fishing coves or rooftops at the city of Coquimbo. This supports the idea that wild gulls follow seasonal peaks in resource abundance and availability and relocate to more profitable areas to avoid deteriorating environmental conditions once they are not constrained by breeding (Whittington et al. 2009). Afterwards, during the austral spring, which encompasses the pre‐breeding period, wild gulls returned to their nesting colonies. This behaviour is consistent with observations of Kelp gulls defending and preserving territories prior to the onset of the breeding season (Chávez‐Villavicencio 2014; Quintana and Yorio 1998).

### 4.4 Management and Conservation Implications

Although based on a limited small sample size, our results not only provide baseline information on habitat use and movement patterns of Kelp gulls, but also suggest that natural resources alone may no longer be sufficient to support Kelp gull populations throughout the year. Globally, several populations of large gulls are already in decline (Langlois Lopez et al. 2023; Ouled‐Cheikh et al. 2021; Serré et al. 2022; Washburn et al. 2016). The apparent rise in urban gull abundance is thought to reflect a behavioural response to decreasing natural prey availability in their traditional habitats, with anthropogenic resources now playing a role in supporting populations (Pierotti and Annett 2001; Serré et al. 2022; Washburn et al. 2016; Whittington et al. 2016). Although improved waste management is essential for public health, it can unintentionally reduce food availability for Kelp gulls, and this decrease in food availability may lead to decreased nutritional intake, fewer breeding pairs, and reduced breeding success, which could eventually lead to sharp population declines (Galbraith et al. 2015; Washburn et al. 2016). For instance, the closure of refuse tips has been linked to the decline of a long‐established Kelp gull colony in New Zealand (Galbraith et al. 2015). Given that both urban and wild Kelp gulls in Chile rely on anthropogenic habitats during at least a part of their life cycle, any management actions that limit their access to food resources could potentially impact both urban and wild populations, and thus, should be implemented with caution.

## Author Contributions

**Miriam Lerma:** conceptualization (equal), data curation (equal), formal analysis (equal), funding acquisition (equal), resources (equal), validation (equal), visualization (equal), writing – original draft (equal), writing – review and editing (equal). **Mylene Seguel:** methodology (equal), writing – original draft (equal), writing – review and editing (equal). **Claudia E. Fernández:** conceptualization (equal), methodology (equal), validation (equal), writing – review and editing (equal). **Stefan Garthe:** conceptualization (equal), project administration (equal), resources (equal), writing – review and editing (equal). **Guillermo Luna‐Jorquera:** resources (equal), writing – review and editing (equal).

## Disclosure

*Statement of inclusion*: Our study brings together authors from a number of different countries, including scientists based in the country where the study was carried out.

## Ethics Statement

Fieldwork was performed following ethical international standards for the care and use of wild animals from Ministerio de Agricultura (SAG), resolución exenta No. 28/2022, and approved by the Universidad Católica del Norte's Scientific Ethics Committee by resolution CEC UCN No. 46.

## Conflicts of Interest

The authors declare no conflicts of interest.

## Supporting information


**Data S1:** ece372974‐sup‐0001‐Supinfo.doc.

## Data Availability

Data used in the analyses are available at movebank (ID: 2491228306). Scripts to reproduce the analyses are available at: https://github.com/MiriamLL/larus.

## References

[ece372974-bib-0001] Adami, M. A. , M. Bertelloti , M. L. Agüero , M. G. Frixione , and V. L. D'Amico . 2024. “Assessing the Impact of Urban Landfills as Feeding Sites on Physiological Parameters of a Generalist Seabird Species.” Marine Pollution Bulletin 202: 116327. 10.1016/j.marpolbul.2024.116327. 38581734

[ece372974-bib-0002] Balaguera‐Quintero, A. , A. Vallone , and S. Igor‐Tapia . 2022. “Carbon Footprint Estimation for La Serena‐Coquimbo Conurbation Based on Global Protocol for Community‐Scale Greenhouse Gas Emission Inventories (GPC).” Sustainability 14: 10309. 10.3390/su141610309.

[ece372974-bib-0003] Banegas Vizuete, G. , E. Cortés Pizarro , and O. Fosado Téllez . 2018. “Plan de Manejo de Residuos de Pescado Para el Puerto Pequero Artesanal de Coquimbo.” Revista de Las Agrociencias 19: 91–114. 10.52052/issn.2176-5960.pro.v9i19.4796.

[ece372974-bib-0004] Bates, D. , M. Mächler , B. M. Bolker , and S. C. Walker . 2015. “Fitting Linear Mixed‐Effects Models Using lme4.” Journal of Statistical Software 67: 1–48. 10.18637/jss.v067.i01.

[ece372974-bib-0005] Bertellotti, M. , and P. Yorio . 1999. “Spatial and Temporal Patterns in the Diet of the Kelp Gull in Patagonia.” Condor 101: 790–798. 10.2307/1370066.

[ece372974-bib-0006] Canti, S. , P. González , N. Suárez , P. Yorio , and C. Marinao . 2023. “Interactions Between Breeding Gulls and Monofilament Lines at One of the Main Recreational Fishing Sites in Argentina.” Marine Pollution Bulletin 188: 114720. 10.1016/j.marpolbul.2023.114720. 36860016

[ece372974-bib-0007] Chávez‐Villavicencio, C. 2014. “Approach to Breeding Site Selection of Kelp Gulls (*Larus dominicanus* Lichtenstein 1832) in an Urban Area From Coquimbo Region (Chile) and a New Nesting Substrate.” Biologist 12: 33–44. 10.24039/rtb2014121384.

[ece372974-bib-0008] Clements, S. J. , B. M. Ballard , G. R. Eccles , E. A. Sinnott , and M. D. Weegman . 2021. “Trade‐Offs in Performance of Six Lightweight Automated Tracking Devices for Birds.” Journal of Field Ornithology 92: 506–517. 10.1111/jofo.12392.

[ece372974-bib-0009] Croxall, J. P. , S. H. M. Butchart , B. E. N. Lascelles , et al. 2012. “Seabird Conservation Status and Threats: A Global Assessment of Priorities.” Bird Conservation International 22: 1–34. 10.1017/S0959270912000020.

[ece372974-bib-0010] Fernández, C. E. , G. Luna‐Jorquera , V. González Encinas , et al. 2024. “Seabirds as Biovectors in the Transport of Plastic Debris Across Ecosystem Borders: A Case Study From the Humboldt Current Upwelling System.” Science of the Total Environment 952: 175938. 10.1016/j.scitotenv.2024.175938. 39218118

[ece372974-bib-0011] Flack, A. , W. Fiedler , J. Blas , et al. 2016. “Costs of Migratory Decisions: A Comparison Across Eight White Stork Populations.” Science Advances 2: e1500931. 10.1126/sciadv.1500931. 26844294 PMC4737271

[ece372974-bib-0012] Foley, M. , K. A. Lato , M. Fuirst , R. R. Veit , R. M. Cerrato , and L. H. Thorne . 2025. “Spatial and Temporal Predictability Drive Foraging Movements of Coastal Birds.” Movement Ecology 13: 5. 10.1186/s40462-025-00531-y. 39893488 PMC11787743

[ece372974-bib-0013] Fox, J. , and S. Weisenberg . 2024. “car: Companion to Applied Regression.” 10.32614/CRAN.package.car.

[ece372974-bib-0014] Frixione, M. G. , N. Lisnizer , and P. Yorio . 2023. “Year‐Round Use of Anthropogenic Food Sources in Human Modified Landscapes by Adult and Young Kelp Gulls.” Food Webs 35: e00274. 10.1016/j.fooweb.2023.e00274.

[ece372974-bib-0015] Galbraith, M. , S. J. Bury , D. L. Fraser , and M. J. Rayner . 2025. “The Karoro *Larus dominicanus* in Northern Aotearoa, New Zealand: Diet and Evidence of Changing Trophic Position From Regurgitated Pellets and Stable Isotope Analysis of Contemporary and Historic Feathers and Bones.” New Zealand Journal of Ecology 49: 3597. 10.20417/nzjecol.49.3597.

[ece372974-bib-0016] Galbraith, M. , J. Krzyzosiak , G. Aguilar , G. Jones , and R. Oliver . 2015. “Changes in the Breeding Status of the Southern Black‐Backed Gull (*Larus dominicanus*) Colonies on Rangitoto Island, Hauraki Gulf, New Zealand.” Notornis 62: 192–201.

[ece372974-bib-0017] Ghersi, B. M. , D. L. Blazes , E. Icochea , et al. 2009. “Avian Influenza in Wild Birds, Central Coast of Peru.” Emerging Infectious Diseases 15: 935–938. 10.3201/eid1506.080981. 19523296 PMC2727326

[ece372974-bib-0018] Giaccardi, M. , and P. Yorio . 2004. “Temporal Patterns of Abundance and Waste Use by Kelp Gulls (*Larus dominicanus*) at an Urban and Fishery Waste Site in Northern Coastal Patagonia, Argentina.” Ornitologia Neotropical 15: 93–102.

[ece372974-bib-0019] Hamer, K. C. , E. A. Schreiber , and J. Burger . 2002. “Breeding Biology, Life Histories, and Life History‐Environment Interactions in Seabirds.” In Biology of Marine Birds, 217–261. CRS Press.

[ece372974-bib-0020] Harting, F. 2024. “DHARMa: Residual Diagnostics for Hierarchical (Multi‐Level/Mixed) Regression Models.” https://CRAN.R‐project.org/package=DHARMa.

[ece372974-bib-0021] Hernández, S. , M. T. González , J. C. Villarroel , and E. Acuña . 2010. “Seasonal Variation in Fish Bycatch Associated With an Artisanal Flounder Fishery on Coquimbo Bay, Chile.” Revista de Biología Marina y Oceanografía 45: 695–703. 10.4067/s0718-19572010000400013.

[ece372974-bib-0022] Hothorn, T. , F. Bretz , P. Westfall , R. M. Heiberger , A. Schuetzenmeister , and S. Scheibe . 2023. “Package Multcomp.” 10.32614/CRAN.package.multcomp.

[ece372974-bib-0023] Kasinsky, T. , N. Suárez , C. Marinao , and P. Yorio . 2018. “Kelp Gull (*Larus dominicanus*) Use of Alternative Feeding Habitats at the Bahía San Blas Protected Area, Argentina.” Waterbirds 41: 285–294. 10.1675/063.041.0308.

[ece372974-bib-0024] Langley, L. P. , S. Bearhop , N. H. K. Burton , et al. 2022. “Urban and Coastal Breeding Lesser Black‐Backed Gulls (*Larus fuscus*) Segregate by Foraging Habitat.” Ibis 165: 214–230. 10.1111/ibi.13109.

[ece372974-bib-0025] Langlois Lopez, S. , A. L. Bold , N. J. O'Hanlon , et al. 2023. “Global Population and Conservation Status of the Great Black‐Backed Gull *Larus Marinus* .” Bird Conservation International 33: e23. 10.1017/S0959270922000181.

[ece372974-bib-0026] Lenzi, J. , I. González‐Bergonzoni , E. Machín , B. Pijanowski , and E. Flaherty . 2019. “The Impact of Anthropogenic Food Subsidies on a Generalist Seabird During Nestling Growth.” Science of the Total Environment 687: 546–553. 10.1016/j.scitotenv.2019.05.485. 31216509

[ece372974-bib-0027] Lerma, M. 2025. “Package Larus.” A R Package to Identify Trips and Assign Habitat Use to Tracking Locations Collected From GSM‐GPS Devices. https://github.com/MiriamLL/larus.

[ece372974-bib-0028] Lisnizer, N. , P. Garcia‐Borboroglu , and P. Yorio . 2011. “Spatial and Temporal Variation in Population Trends of Kelp Gulls in Northern Patagonia, Argentina.” Emu 111: 259–267. 10.1071/MU11001.

[ece372974-bib-0029] Lüdecke, D. 2024. “Package ‘Performance.’” R Package. 10.1098/rsif.2017.0213.

[ece372974-bib-0030] Ludynia, K. , S. Garthe , and G. Luna‐Jorquera . 2005. “Seasonal and Regional Variation in the Diet of the Kelp Gull in Northern Chile.” Waterbirds 28: 359–365. 10.1675/1524-4695(2005)028[0359:SARVIT]2.0.CO;2.

[ece372974-bib-0031] Marinao, C. , T. Kasinsky , N. Suárez , and P. Yorio . 2018. “Contribution of Recreational Fisheries to the Diet of the Opportunistic Kelp Gull.” Austral Ecology 43: 861–875. 10.1111/aec.12627.

[ece372974-bib-0032] McGillycuddy, M. , D. I. Warton , G. Popovic , and B. M. Bolker . 2025. “Parsimoniously Fitting Large Multivariate Random Effects in glmmTMB.” Journal of Statistical Software 112: 1–19. 10.18637/jss.v112.i01.

[ece372974-bib-0033] McKinney, M. L. , and J. L. Lockwood . 1999. “Biotic Homogenization: A Few Winners Replacing Many Losers in the Next Mass Extinction.” Trends in Ecology & Evolution 14: 450–453. 10.1016/S0169-5347(99)01679-1. 10511724

[ece372974-bib-0034] Navarro, J. , D. Grémillet , F. J. Ramirez , I. Afán , W. Bouten , and M. G. Forero . 2017. “Shifting Individual Habitat Specialization of a Successful Predator Living in Anthropogenic Landscapes.” Marine Ecology Progress Series 578: 243–251. 10.3354/meps12124.

[ece372974-bib-0035] O'Hanlon, N. J. , C. B. Thaxter , N. H. K. Burton , et al. 2022. “Habitat Selection and Specialisation of Herring Gulls During the Non‐Breeding Season.” Frontiers in Marine Science 9: 816881. 10.3389/fmars.2022.816881.

[ece372974-bib-0036] Orellana McBride, A. G. 2020. “Metropolitan Formation From Fragmentation the Conurbation Process of Greater La Serena.” Urbano 41: 58–83. 10.22320/07183607.2020.23.41.04.

[ece372974-bib-0037] Oro, D. , M. Genovart , G. Tavecchia , M. S. Fowler , and A. Martínez‐Abraín . 2013. “Ecological and Evolutionary Implications of Food Subsidies From Humans.” Ecology Letters 16: 1501–1514. 10.1111/ele.12187. 24134225

[ece372974-bib-0038] Ouled‐Cheikh, J. , V. Morera‐Pujol , Á. Bahillo , F. Ramírez , M. Cerdà‐Cuéllar , and R. Ramos . 2021. “Foraging in the Anthropocene: Feeding Plasticity of an Opportunistic Predator Revealed by Long Term Monitoring.” Ecological Indicators 129: 107943. 10.1016/j.ecolind.2021.107943.

[ece372974-bib-0039] Pastén‐Araya, J. , C. E. Fernández , M. Seguel , N. Luna , and G. Luna‐Jorquera . 2021. “The Kelp Gull (*Larus dominicanus*) Preys Upon Chicks of Peruvian Diving‐Petrels (*Pelecanoides garnotii*) in Choros Island, Northern Chile.” Revista Chilena de Ornitología 27: 37–40.

[ece372974-bib-0040] Pereda, A. J. , M. Uhart , A. A. Perez , et al. 2008. “Avian Influenza Virus Isolated in Wild Waterfowl in Argentina: Evidence of a Potentially Unique Phylogenetic Lineage in South America.” Virology 378: 363–370. 10.1016/j.virol.2008.06.010. 18632129 PMC2570041

[ece372974-bib-0041] Pierotti, R. , and C. Annett . 2001. “The Ecology of Western Gulls in Habitats Varying in Degree of Urban Influence.” In Avian Ecology and Conservation in an Urbanizing World, 307–329. Springer. 10.1007/978-1-4615-1531-9_15.

[ece372974-bib-0042] Plaza, P. I. , and S. A. Lambertucci . 2017. “How Are Garbage Dumps Impacting Vertebrate Demography, Health, and Conservation?” Global Ecology and Conservation 12: 9–20. 10.1016/j.gecco.2017.08.002.

[ece372974-bib-0043] Prellvitz, L. J. , R. I. Hogan , and C. M. Vooren . 2009. “Breeding Biology of Kelp Gulls (*Larus dominicanus*) on Deserta Island, Southern Brazil.” Ornitología Neotropical 20: 61–72.

[ece372974-bib-0044] Quintana, F. , and P. Yorio . 1998. “Competition for Nest Sites Between Kelp Gulls (*Larus dominicanus*) and Terns (*Sterna maxima* and *S. eurygnatha*) in Patagonia.” Auk 115: 1068–1071. 10.2307/4089525.

[ece372974-bib-0045] Ramírez, F. , I. Afán , W. Bouten , J. L. Carrasco , M. G. Forero , and J. Navarro . 2020. “Humans Shape the Year‐Round Distribution and Habitat Use of an Opportunistic Scavenger.” Ecology and Evolution 10: 4716–4725. 10.1002/ece3.6226. 32551055 PMC7297764

[ece372974-bib-0046] Reusch, K. , N. Suárez , P. G. Ryan , and L. Pichegru . 2020. “Foraging Movements of Breeding Kelp Gulls in South Africa.” Movement Ecology 8: 36. 10.1186/s40462-020-00221-x. 32905351 PMC7469291

[ece372974-bib-0047] Rodewald, A. D. , and D. P. Shustack . 2008. “Consumer Resource Matching in Urbanizing Landscapes: Are Synanthropic Species Over‐Matching?” Ecology 89: 515–521. 10.1890/07-0358.1. 18409440

[ece372974-bib-0048] Rodríguez, F. , J. Moreno , R. Ortega , et al. 2012. “Evidence for Kelp Gulls (*Larus dominicanus*) and Franklin's Gulls (*Leucophaeus pipixcan*) as Carriers of Salmonella by Real‐Time Polymerase Chain Reaction.” Journal of Wildlife Diseases 48: 1105–1108. 10.7589/2012-04-104. 23060519

[ece372974-bib-0049] Serré, S. , C. Irvine , K. Williams , and C. E. Hebert . 2022. “Lake Superior Herring Gulls Benefit From Anthropogenic Food Subsidies in a Prey‐Impoverished Aquatic Environment.” Journal of Great Lakes Research 48: 1258–1269. 10.1016/j.jglr.2022.08.008.

[ece372974-bib-0050] Silva‐Costa, A. , and L. Bugoni . 2013. “Feeding Ecology of Kelp Gulls (*Larus dominicanus*) in Marine and Limnetic Environments.” Aquatic Ecology 47: 211–224. 10.1007/s10452-013-9436-1.

[ece372974-bib-0051] Simeone, A. , and G. Luna‐Jorquera . 2012. “Estimating Rat Predation on Humboldt Penguin Colonies in North‐Central Chile.” Journal of Ornithology 153: 1079–1085. 10.1007/s10336-012-0837-z.

[ece372974-bib-0052] Simeone, A. , G. Luna‐Jorquera , M. Bernal , et al. 2003. “Breeding Distribution and Abundance of Seabirds on Islands Off Northcentral Chile.” Revista Chilena de Historia Natural 76: 323–333. 10.4067/S0716-078X2003000200016.

[ece372974-bib-0053] Soriano‐Redondo, A. , A. M. A. Franco , M. Acácio , B. H. Martins , F. Moreira , and I. Catry . 2021. “Flying the Extra Mile Pays‐Off: Foraging on Anthropogenic Waste as a Time and Energy‐Saving Strategy in a Generalist Bird.” Science of the Total Environment 782: 146843. 10.1016/j.scitotenv.2021.146843.

[ece372974-bib-0054] Souc, C. , C. Leray , T. Blanchon , et al. 2024. “No Detrimental Effects of Wing‐Harnessed GPS Devices on the Breeding Performance of Yellow‐Legged Gulls (*Larus michahellis*): A Multi‐Colony Evaluation.” Ibis 166: 1404–1412. 10.1111/ibi.13338.

[ece372974-bib-0055] Steele, W. K. 1992. “Diet of Hartlaub's Gull *Larus hartlaubii* and the Kelp Gull *L. dominicanus* in the Southwestern Cape Province, South Africa.” Ostrich 63: 68–82. 10.1080/00306525.1992.9633952.

[ece372974-bib-0056] Svagelj, W. S. , N. Lisnizer , P. G. Borboroglu , and P. Yorio . 2015. “Variation in the Size of Eggs of Kelp Gulls (*Larus dominicanus*) at Two Colonies in Patagonia, Argentina.” Waterbirds 38: 92–98. 10.1675/063.038.0112.

[ece372974-bib-0057] Thaxter, C. B. , V. H. Ross‐Smith , J. A. Clark , et al. 2014. “A Trial of Three Harness Attachment Methods and Their Suitability for Long‐Term Use on Lesser Black‐Backed Gulls and Great Skuas.” Ringing & Migration 29: 65–76. 10.1080/03078698.2014.995546.

[ece372974-bib-0058] Thiel, M. , E. Macaya , E. Acuña , et al. 2007. “The Humboldt Current System of Northern and Central Chile.” Oceanography and Marine Biology: An Annual Review 45: 195–344. 10.1201/9781420050943.ch6.

[ece372974-bib-0059] Villablanca, R. , G. Luna‐Jorquera , V. H. Marín , S. Garthe , and A. Simeone . 2007. “How Does a Generalist Seabird Species Use Its Marine Habitat? The Case of the Kelp Gull in a Coastal Upwelling Area of the Humboldt Current.” ICES Journal of Marine Science 64: 1348–1355. 10.1093/icesjms/fsm120.

[ece372974-bib-0060] Washburn, B. E. , S. B. Elbin , and C. Davis . 2016. “Historical and Current Population Trends of Herring Gulls (*Larus argentatus*) and Great Black‐Backed Gulls (*Larus marinus*) in the New York Bight, USA.” Waterbirds 39: 74–86. 10.1675/063.039.sp114.

[ece372974-bib-0061] Whittington, P. A. , R. J. M. C. Ford , A. P. Martin , et al. 2016. “Recent Trends of the Kelp Gull (*Larus dominicanus*) in South Africa.” Waterbirds 39: 99–113. 10.1675/063.039.sp102.

[ece372974-bib-0062] Whittington, P. A. , A. Paul Martin , N. T. W. Klages , and A. Schultz . 2009. “Movements of the Kelp Gull *Larus dominicanus vetula* To, From and Within Southern South Africa.” Marine Ornithology 37: 139–152.

[ece372974-bib-0063] Yoda, K. , N. Tomita , Y. Mizutani , A. Narita , and Y. Niizuma . 2012. “Spatio‐Temporal Responses of Black‐Tailed Gulls to Natural and Anthropogenic Food Resources.” Marine Ecology Progress Series 466: 249–259. 10.3354/meps09939.

[ece372974-bib-0064] Yorio, P. , J. O. Branco , J. Lenzi , G. Luna‐Jorquera , and C. Zavalaga . 2016. “Distribution and Trends in Kelp Gull (*Larus dominicanus*) Coastal Breeding Populations in South America.” Waterbirds 39: 114–135. 10.1675/063.039.sp103.

[ece372974-bib-0065] Yorio, P. , and P. García Borboroglu . 2002. “Breeding Biology of Kelp Gulls (*Larus dominicanus*) at Golfo San Jorge, Patagonia, Argentina.” Emu 102: 257–263. 10.1071/MU00077.

[ece372974-bib-0066] Yorio, P. , C. Marinao , and N. Suárez . 2014. “Kelp Gulls ( *Larus dominicanus* ) Killed and Injured by Discarded Monofilament Lines at a Marine Recreational Fishery in Northern Patagonia.” Marine Pollution Bulletin 85: 186–189. 10.1016/j.marpolbul.2014.05.052. 24951250

[ece372974-bib-0067] Yorio, P. , C. Marinao , N. Suárez , et al. 2024. “Use of Anthropogenic Resources by Seabirds Breeding in Argentine Patagonia.” Hornero 39: 139–172. 10.56178/eh.v39i2.1491.

