# Supplementary material for: Urban and Wild Kelp Gulls: Tracking Seasonal Shifts in Habitat Use and Movement Patterns
Source: Ecol Evol. 2026 Jan 20;16(1):e72974. doi: 10.1002/ece3.72974 (PMC12819176; doi:10.1002/ece3.72974)
Supplement: Supplementary file 1 — Data S1: ece372974‐sup‐0001‐Supinfo.doc. [file ECE3-16-e72974-s001.doc]

# **Supplementary materials**

## **Figure S1.** Proportion of habitat sites used by urban (in red) and wild (in blue) Kelp gulls separated by Austral summer (December, January, February), autumn (March, April, May), winter (June, July, August), and spring (September, October, November). Habitat use was calculated as the proportion of locations in each habitat type grouped by the corresponding period. Box plots show the interquartile range (IQR) across individuals; the horizontal line indicating the median; whiskers extend to 1.5 times the IQR; points represent outliers.





## **Table S1.** Movement parameters of urban and wild Kelp gulls during the austral seasons in Northern Chile. Trip duration is the time between departure and arrival to the colony or resting location. Maximum distance is the farthest point from the colony per trip. Path length is the total distance travelled. Values are presented as median and 95% confidence intervals (CIs). Care should be taken when interpreting this results, as more trips were identified in summer (n = 1281) and autumn (n = 847) than in winter (n = 292) and spring (n = 519).

|  | Summer | Autumn | Winter | Spring |
| --- | --- | --- | --- | --- |
| **Trip duration (h)** |  |  |  |  |
| Urban gulls | 0.8 [95% CI: 0.6–1.3] | 1.9 [95% CI: 1.0–3.4] | 5.3 [95% CI: 4.0–12.0] | 1.3 [95% CI: 1.0–2.0] |
| Wild gulls | 2.7 [95% CI: 1.6–5.8] | 7.9 [95% CI: 2.2–12.1] | 10.0 [95% CI: 4.7–12.7] | 9.7 [95% CI: 2.9–12.2] |
| **Maximum distance (km)** |  |  |  |  |
| Urban gulls | 5.7 [95% CI: 0.6–5.8] | 5.1 [95% CI: 0.6–5.7] | 1.4 [95% CI: 0.6–5.6] | 5.6 [95% CI: 4.2–5.7] |
| Wild gulls | 8.1 [95% CI: 7.3–29.4] | 29.7 [95% CI: 8.2–71.3] | 88.0 [95% CI: 32.5–92.3] | 25.5 [95% CI: 7.3–85.4] |
| **Path length (km)** |  |  |  |  |
| Urban gulls | 11.7 [95% CI: 1.6–16.3] | 11.6 [95% CI: 1.7–16.0] | 3.6 [95% CI: 1.4–12.3] | 11.5 [95% CI: 11.1–16.3] |
| Wild gulls | 20.4 [95% CI: 16.4–40.9] | 17.4 [95% CI: 3.7–30.6] | 27.9 [95% CI: 10.7–71.8] | 17.9 [95% CI: 7.5–67.6] |

**Trip duration:** Urban gulls consistently made shorter trips than wild gulls across seasons, particularly during summer (Tukey HSD test p < 0.01); however differences between spring and autumn were not statistical significant (Tukey HSD test p > 0.05). **Maximum distance:** Urban gulls staying closer to their colonies during winter, and wild gulls during summer (Tukey HSD test, p < 0.01); however differences between spring and autumn were not statistical significant (Tukey HSD test p > 0.05). **Path length:** Urban gulls covered shorter path lengths in winter and wild gulls in autumn (Tukey HSD test, p < 0.01); however, differences between winter and spring, as well as between winter and summer, were not statistical significant (Tukey HSD test p > 0.05).
